# Genotypic and Antimicrobial Characterisation of *Propionibacterium acnes* Isolates from Surgically Excised Lumbar Disc Herniations

**DOI:** 10.1155/2013/530382

**Published:** 2013-08-28

**Authors:** Jess Rollason, Andrew McDowell, Hanne B. Albert, Emma Barnard, Tony Worthington, Anthony C. Hilton, Ann Vernallis, Sheila Patrick, Tom Elliott, Peter Lambert

**Affiliations:** ^1^The School of Health and Life Sciences, Department of Biomolecular Sciences, Coventry University, CV1 5FB, UK; ^2^Centre for Infection and Immunity, School of Medicine, Dentistry and Biomedical Sciences, Queen's University, Belfast BT9 7BL, UK; ^3^Research Department, Spine Centre of Southern Denmark, Hospital Lillebaelt, Middelfart, Institute of Regional Health Services Research, University of Southern Denmark, 5230, Denmark; ^4^The School of Life and Health Sciences, Aston University, Birmingham B4 7ET, UK; ^5^University Hospital NHS Trust, Birmingham B15 2TH, UK

## Abstract

The anaerobic skin commensal *Propionibacterium acnes* is an underestimated cause of human infections and clinical conditions. Previous studies have suggested a role for the bacterium in lumbar disc herniation and infection. To further investigate this, five biopsy samples were surgically excised from each of 64 patients with lumbar disc herniation. *P. acnes* and other bacteria were detected by anaerobic culture, followed by biochemical and PCR-based identification. In total, 24/64 (38%) patients had evidence of *P. acnes* in their excised herniated disc tissue. Using *recA* and mAb typing methods, 52% of the isolates were type II (50% of culture-positive patients), while type IA strains accounted for 28% of isolates (42% patients). Type III (11% isolates; 21% patients) and type IB strains (9% isolates; 17% patients) were detected less frequently. The MIC values for all isolates were lowest for amoxicillin, ciprofloxacin, erythromycin, rifampicin, tetracycline, and vancomycin (≤1mg/L). The MIC for fusidic acid was 1-2 mg/L. The MIC for trimethoprim and gentamicin was 2 to ≥4 mg/L. The demonstration that type II and III strains, which are not frequently recovered from skin, predominated within our isolate collection (63%) suggests that the role of *P. acnes* in lumbar disc herniation should not be readily dismissed.

## 1. Introduction

As a member of the normal skin and oral microbiota, *Propionibacterium acnes* (*P. acnes*) is frequently dismissed as a contaminant of clinical samples. While a likely pathogenic role in the inflammatory skin condition acne vulgaris has been well documented [1], evidence is now emerging that suggests *P. acnes* is important in a number of other infections and clinical conditions. These include infections of indwelling medical devices [2], sciatica [3], and discitis [4, 5]. Furthermore, whole genome sequencing of multiple *P. acnes* strains has provided valuable insights into the range of putative virulence factors and proteins that may facilitate attachment, inflammation, and pathogenicity [6, 7].

As a consequence of tooth brushing and endodontic therapy, *P. acnes* can asymptomatically invade the blood stream leading to transient bacteraemia that may be important in the initiation of particular types of infection [8, 9]. A previous study has suggested an association between *P. acnes* and lumbar disc herniation [3]. Intervertebral disc herniation causes nuclear disc material to be forced into the spinal canal where neocapillarisation occurs around the tissue followed by accumulation of mononuclear leukocytes, macrophages, and inflammation [10, 11]. Although *P. acnes* may be present in low numbers in the bloodstream, the avascular nuclear disc material in the spinal canal could provide an ideal anaerobic environment for opportunistic *P. acnes* infection [12]. It has therefore been proposed that *P. acnes* may cause chronic, low-grade infection in herniated discs. Such infection in patients with lumbar disc herniation could lead to bone oedema (Modic changes/low grade discitis), low back pain, and inflammation [12]. Recent studies suggest discs infected with anaerobic bacteria are more likely to develop Modic changes and low grade discitis in the adjacent vertebrae than those in which no bacteria or aerobic bacteria are isolated [13]. Distinguishing between a bacterial association with lumbar disc herniation and contamination of the surgical wound by the normal skin microbiota is critical in defining a microbial role [14]. Research has demonstrated that antibiotic treatment has a positive effect upon physiological symptoms and reducing pain associated with lumbar disc herniation and chronic back conditions [15, 16]. This observation, along with evidence that *P. acnes* can be isolated from excised disc herniation tissue, supports the theory that bacterial infection may play a key role [13]. Molecular analysis of *P. acnes* isolated from disc herniation tissue is now essential to further understand this condition.

We have previously shown that *P. acnes* comprises a number of distinct evolutionary lineages, designated types IA, IB, II, and III, based on single locus phylotyping [17, 18]. This classification has now been expanded based on Multilocus Sequence Typing (MLST) and whole genome sequencing to types IA_1_, IA_2_, IB, IC, II, and III which is further supported at the clinical and phenotypic level [19]. Evidence suggests these phylogroups may have differing pathogenicity traits and may be associated with various clinical disease states [20–22]. In particular, type III strains have been linked to surgically excised spinal disc material, although it is unclear at this stage if this is clinically meaningful [18].

The aim of this study was to investigate the phylogroup status of *P. acnes* cultured from excised disc nucleus material of 64 patients with lumbar disc herniations. In addition, antimicrobial susceptibilities were determined to inform possible future treatment regimens and studies.

## 2. Materials and Methods

### 2.1. Study Subjects and Biopsy Collection

A total of 64 patients (attending The Mølholm Hospital, Vejle, Denmark) undergoing discectomy surgery, and between the ages of 18–65 years, were included in this study [13]. All patients were diagnosed with lumbar disc herniation confirmed by MRI. Patients were excluded if antibiotic treatment had been received within two weeks of the study start date. All patients were immunocompetent and none had received a previous epidural steroid injection or undergone back surgery. Five separate nucleus disc tissue samples were surgically removed from each patient as recommended by the IDSA for investigation of prosthetic joint infection [23]. To reduce any potential contamination of excised biopsies, skin was treated preoperatively for 2 min with 2% (v/v) chlorhexidine in 70% (v/v) isopropyl alcohol. The nucleus material (centre of the disc that is herniated) was extracted with a fresh set of sterile instruments for each individual biopsy. To prevent growth inhibition of any bacteria present in the biopsy samples, one high dose (1.5 g) cefuroxime was administered intravenously after the tissue samples were retrieved. Biopsies were placed in separate sterile glass vials and immediately frozen at −80°C. The samples were transported to Aston University frozen in thermal transport boxes designed for organ transport.

### 2.2. Bacterial Culture

All tissue samples were cut into smaller fragments, and the tissue broken apart and ground up, using an individually packaged sterile scalpel [3]. As a further precaution, all scalpels were dipped in 70% (v/v) ethanol and passed through a Bunsen burner flame before use. With a sterilised scalpel, the processed and ground up tissue sample was first spread across the surface of a Columbia blood agar (CBA) plate (Oxoid, UK), and then collectively embedded into the centre of the plate [3]. For each individual tissue sample, one section of tissue was used for aerobic and one for anaerobic incubation at 37°C (Don Whitley MiniMacs Anaerobic Workstation, 80% nitrogen, 10% CO_2_ and 10% H_2_). Plates were incubated for a minimum of seven days.

### 2.3. Molecular and Phenotypic Identification

Colonies were subcultured onto CBA plates and incubated under aerobic or anaerobic conditions for 24 hrs at 37°C before Gram-staining. Presumptive *P. acnes* isolates were then identified by biochemical analysis using the Rapid ID 32A kit (bioMerieux). Presumptive *Staphylococcus *spp. were identified using standard biochemical tests (oxidase and catalase), and latex agglutination for clumping factor/protein A used to distinguish *S. aureus* from coagulase-negative staphylococci. *P. acnes* isolates were confirmed by 16S rRNA-based PCR using the primers and conditions previously described [24] after extraction of genomic DNA using a rapid boil method [25]. 

### 2.4. *recA* Sequence Analysis

Nucleotide sequence analysis of the *recA* housekeeping gene was initially used to differentiate *P. acnes* isolates into phylogroups IA, IA/IB, IC, II, or III [17, 18]. The *recA* locus was amplified with the previously described primers PAR-1 and PAR-2, which are directed to downstream and upstream flanking sequences of the *recA* open reading frame, respectively, and generate a 1201 bp amplicon [17]. Sequencing reactions were performed using ABI PRISM ready reaction terminator cycle sequencing kits (version 1.1; Perkin-Elmer Applied Biosystems) according to the manufacturer's instructions and the samples analysed on an ABI PRISM 3100 genetic analyser capillary electrophoresis system (Perkin-Elmer Applied Biosystems). 

### 2.5. Monoclonal Antibody Typing

Monoclonal antibody (mAb) typing by immunofluorescence microscopy (IFM) was carried out as previously described [17]. All *P. acnes* isolates were examined with the mouse mAbs QUBPa1 and QUBPa2, which target dermatan-sulphate-binding adhesins and a carbohydrate/glycolipid-containing antigen on type IA and type II strains, respectively [17, 18]. Strains of type IB or type III do not react with these mAbs, while all type IC isolates analysed to date show dual reaction in keeping with their distinct nature [26]. Slides were viewed using a Leitz Dialux 20 fluorescence microscope.

### 2.6. Minimum Inhibitory Concentration (MIC) Determination by Agar Dilution

The MIC was determined for each isolate against a range of antibiotics using the CLSI reference agar dilution procedure [27]. The following antibiotics were used: amoxicillin, erythromycin, tetracycline, trimethoprim, fusidic acid, gentamicin, rifampicin, vancomycin, and ciprofloxacin (Mast Diagnostics, UK). *P. acnes* type strain NCTC 737 (type IA_1_) was used as a control. Plates were incubated anaerobically for 72 hours at 37°C. The MIC of each antibiotic for each isolate was recorded at the lowest concentration at which there was no visible growth. 

### 2.7. Statistical analysis

Overall phylogroup distribution was analysed using the Chi-squared test. Statistical significance was taken as *P* < 0.05. 

## 3. Results

### 3.1. Culture of *P. acnes* and *Staphylococcus* spp. from Intervertebral Disc Tissue

**Figure 1:**
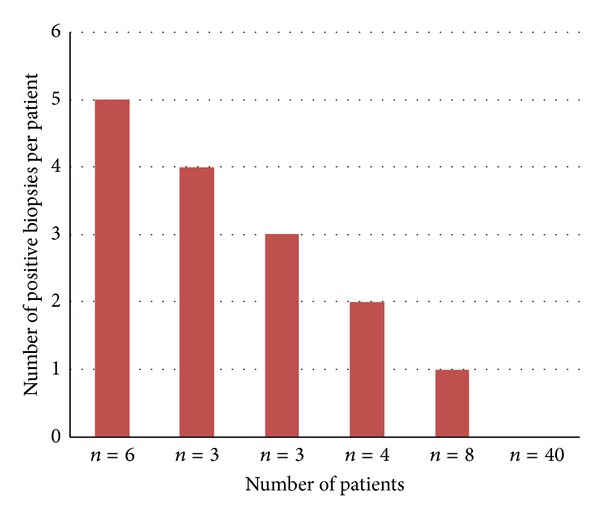
Number of *P. acnes* positive samples recovered from 64 patients undergoing discectomy surgery.**  **Five separate nucleus disc tissue samples were surgically removed from each patient. A total of one or more biopsy specimens recovered from 24 patients were positive for growth of *P. acnes*.

A total of 24/64 (38%) patients were positive for *P. acnes* by culture of their intervertebral disc tissue following discectomy; colony numbers ranged from 1–150 CFU per sample. In addition, we also noted that for all positive samples a lawn of bacterial growth between the embedded tissue and the agar was present, along with a zone of *α*-haemolysis around the tissue. A total of six patients were positive for *P. acnes* in all five of their samples (*n* = 30), while three patients were positive in 4/5 (*n* = 12), three patients in 3/5 (*n* = 9), four patients in 2/5 (*n* = 8) and eight patients in 1/5 (*n* = 8) of their samples (Figure 1). No significant distribution was seen between colonies isolated and number of positive samples per patient. In total, 67 *P. acnes* isolates were recovered from 24 patients. A total of 5/64 (8%) patients were positive for presumptive coagulase-negative *Staphylococcus *spp. In total, only two patients had tissue samples containing both *P. acnes* and coagulase-negative staphylococci. In both cases, the coagulase-negative staphylococci were only present in 1/5 five tissue samples obtained.

Additionally, from one patient (positive for *P. acnes*) a single Gram negative diplococci colony was isolated following aerobic culture. This isolate was identified on only one of the five tissue samples analysed for that patient.

### 3.2. *recA *and mAb Typing of *P. acnes* Isolates

**Figure 2:**
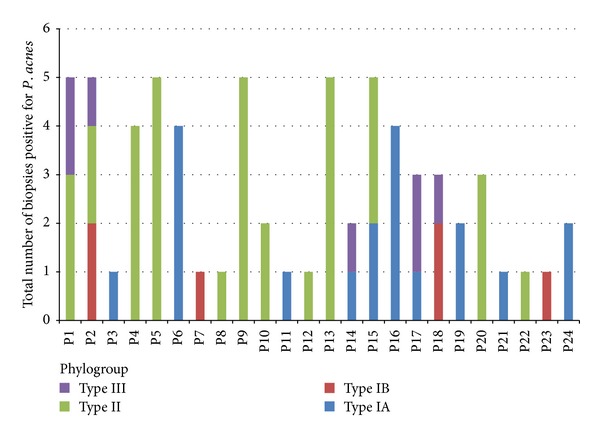
Phylogroup distribution of *P. acnes* isolates recovered from excised disc tissue (*n* = 67). A total of one or more biopsy specimens recovered from 24/64 patients were positive for growth of *P. acnes*. Phylogroups were identified using a combination of *recA *and mAb typing. Five separate nucleus disc tissue samples were surgically removed from each patient as recommended by the IDSA [23]. P = patient number.

Using the combined approach of *recA* sequence analysis and mAb typing, it was possible to accurately classify all *P. acnes* isolates into phylogroups IA (IA_1_ and IA_2_), IB, IC, II, or III. Of the 67 *P. acnes* isolates, 52% were found to belong to the type II lineage, present in 12/24 (50%) patients, 28% were type IA, present in 10/24 (42%) patients, 11% type III, present in 5/24 (21%) patients, and 9% type IB, recovered from 4/24 (17%) patients (*P* < 0.001). No isolates were found to belong to the recently described type IC lineage [26]. In 6/24 patients, multiple *P. acnes* phylogroups were identified from the five tissue samples obtained. These results are illustrated in Figure 2. As part of our epidemiological investigations, we also examined a further seven *P. acnes* isolates recovered from excised disc tissue during a previous study. A total of four isolates were type III, two were type IB and one isolate was type IA. Of all 74 isolates examined, therefore, 48% were type II, 27% type IA, 14% type III, and 11% type IB (*P* < 0.0001).

### 3.3. Antibiotic Susceptibility

For all *P. acnes* isolates tested (including type strain NCTC 737) the MIC values were lowest for amoxicillin, ciprofloxacin, erythromycin, rifampicin, tetracycline, and vancomycin (≤1 mg/L). The MIC for fusidic acid was 1-2 mg/L. The MIC for trimethoprim and gentamicin was 2 to >4 mg/L (Table 1).

## 4. Discussion

In this study, 24/64 (38%) patients with a lumbar disc herniation had *P. acnes* present in intervertebral disc tissue following discectomy. This supports previous findings that also demonstrated the presence of *P. acnes* in excised disc material [3, 28]. Herniated discs reside in an environment of low oxygen tension due to the lack of vascularisation. This may provide an ideal environment for anaerobic bacteria, such as *P. acnes*, to multiply and manifest as a localised infection. The prevalence of *P. acnes* in herniated disc tissue may indicate bacterial infection as a possible cause of the clinical manifestations associated with this condition, such as bone oedema (Modic changes), low back pain and inflammation [12, 15]. To balance this possibility, previous studies have shown that the presence of *P. acnes* in spinal tissue can reflect intraoperative skin contamination [14]. It is important, therefore, to recognise that surgical wound contamination is an important issue during back surgery, even after preoperative skin disinfection, and that biopsy contamination must always be considered. The IDSA state that two or more intraoperative positive cultures that yield the same organism may be considered as evidence of prosthetic joint infection, applying similar criteria to this study suggests infection in 16 (25%) of the 64 patients. Eight patients (12.5%) were positive for *P. acnes* in only 1/5 of their samples; IDSA guidelines indicate these isolates may not represent true infection [23]. This study, and those of others, does however provide some evidence to suggest that the role of intracellular disc bacteria in the pathophysiology of disc degeneration and herniation should not readily be dismissed as contamination, at least not in all contexts [29]. 

In this study, to reduce any possible contamination of the excised biopsies, skin was cleaned preoperatively for 2 min with 2% (v/v) chlorhexidine in 70% (v/v) isopropyl alcohol. Recent studies have demonstrated the efficacy of 2% (v/v) chlorhexidine in 70% (v/v) isopropyl alcohol for skin disinfection prior to bone surgery, where the rate of positive cultures after skin preparation was zero [30]. This provides evidence that such preparations are effective against bacteria present on the skin surface overlying the lumbar spine; however, it is unclear if such methods are as effective against bacteria within deeper parts of the skin. The majority of tissue extracts provided monocultures of *P. acnes* under anaerobic culture, although in six patients multiple phylotypes were present. If skin contamination during surgery or laboratory processing occurred, then we may assume that a greater variety of resident skin microbiota would have been detected. Only two patients had tissue samples containing both *P. acnes* and coagulase-negative staphylococci. In these cases, coagulase-negative staphylococci were only present in 1/5 tissue samples obtained. Furthermore, Stirling et al. [3] used the same skin operating procedures as this study and found that skin contamination was absent in control samples from patients undergoing spinal surgery not related to lumbar disc herniation. 

To investigate the phylogroup pattern of all *P. acnes* isolates we used a combination of previously described *recA* and mAb typing methods [17–19]. The *recA* housekeeping gene has proved a very robust locus for differentiation of strains into the main genetic divisions of *P. acnes* (types I, II, and III) but has been shown to suffer from reduced specificity when it is used to identity some strains of type IA from IB [18]. The latter problem arises as type IA_1_ strains from clonal complex (CC) 4 (Belfast MLST scheme), and all type IA_2_ strains, contain the same *recA* sequence polymorphism that defines all type IB strains [19, 31]. If *recA* sequencing is used, however, in conjunction with mAbs that target type IA and type II strains, then all isolates can be reliably differentiated into types IA, IB, IC, II, and III. The method is relatively straightforward and inexpensive when compared to other techniques, such as MLST, but clearly will not provide high resolution typing of strains if required. Nevertheless, the method still provides valuable epidemiological data and proves extremely useful for prioritising isolates for further downstream analyses.

With this approach we found that type II strains were the dominant phylogroup isolated followed by type IA, and to a lesser extent type III and type IB. These results fit with previous observations that strains of type IB, II, and III, while infrequently isolated from acneic skin, appear more typically associated with blood, soft tissue, medical implant, endodontic infections, and normal skin [17–19, 31–33], although the clinical relevance of these associations remains unclear in many instances. It is important to note that type II and III strains, which collectively represent the majority of all the isolates, do not appear to be especially abundant on the skin when compared with type IA strains, at least based on the analysis of facial and upper trunk skin [17, 31]. This provides further evidence to support the view that their presence in excised disc material should not simply be dismissed as a result of contamination from skin microbiota surrounding the surgical wound, at least not in all cases. Interestingly, the type III lineage was only discovered when isolates from excised disc material were analysed [18]. The observation that type IA strains also represented a significant proportion of the *P. acnes* population isolated from excised disc material is also consistent with previous observations that this evolutionary division of *P. acnes*, while strongly associated with moderate-to-severe cases of acne [19, 31], can also be associated with other types of soft tissue infections, including bacterial keratitis and endophthalmitis [19]. We found no evidence for the recently described multiresistant type IC group amongst our isolates (containing a unique *recA* allele) which, to date, appears only acne associated [26]. While specific *P. acnes* phylogroups have been linked with various clinical conditions and pathogenic lineages [19], it would appear that in the case of lumbar disc herniation, multiple types can still be isolated. While the clinical importance of these different phylogroups is unclear in relation to herniated discs, the particular predominance of type II strains may prove an important observation and tentatively suggest a potential pathogenic role in this condition; in six patients only type II strains were isolated from multiple samples. Further work will, however, be required before any solid conclusions can be made.

Determining the antimicrobial susceptibility of isolates associated with lumbar herniation and discitis may inform future treatment regimes. In this study, we found that susceptibilities were comparable to previous studies with clinical *P. acnes* isolates [34–36]. Treatment of prosthetic or postsurgical *P.  acnes* infection currently includes penicillin/amoxicillin, vancomycin, clindamycin, or rifampicin/linezolid [37–40]. This study demonstrates low MIC values for a number of these commonly used antimicrobials against *P. acnes* isolated in this study. Current treatment for back pain focuses upon pain control, physiotherapy, and surgery. Prevalence of *P. acnes* at the site of ruptured lumbar discs as demonstrated by this study and at the site of sciatica [3] suggests antibiotics may play a future role in the treatment of back pain. Albert et al. [15, 16] demonstrated antibiotic treatment with Amoxicillin-clavulanate had a positive effect upon patients with chronic back conditions in relation to lower back pain intensity, number of days with pain, disease-, and patient-specific function. Demonstration that discs infected with anaerobic bacteria are more likely to develop Modic changes and low grade discitis [13], along with evidence that *P. acnes* can be isolated from excised disc herniation tissue (demonstrated by this study) supports the theory that bacterial infection may play a key role.

Prolonged antimicrobial treatment of acne vulgaris has resulted in the emergence of *P. acnes* strains with resistance to erythromycin and tetracycline [41–43]. Multiresistance is often observed in isolates originating from acneic skin [19]. In contrast, all the type IA strains obtained from disc tissue in this study demonstrate sensitivity to both tetracycline and erythromycin. 

## 5. Conclusion

In this study, a total of 38% of patients had *P. acnes* present at the site of disc herniation. This study therefore provides further evidence to support a possible association between *P. acnes* and chronic low-grade infection in herniated discs, potentially leading to clinical manifestations associated with this condition, such as bone oedema (modic changes), low back pain, and inflammation. While we accept that even after stringent preoperative skin disinfection, contamination of biopsy samples with skin microbiota cannot be fully excluded, the predominance of isolates from lineages not commonly recovered from skin in relative terms does provide tentative evidence in favour for a role in disc herniation. *P. acnes* isolated from excised disc tissue remains susceptible to most classes of antimicrobials, which may prove important for informing future treatment regimens for back pain associated with lumbar disc herniation. While *P. acnes* isolated from herniated discs demonstrated genetic variability between and within individual patient samples, the dominance of type II isolates suggests strains from this particular division might be important in this clinical condition. Large scale studies must now be pursued to definitively determine the importance of *P. acnes* at the site of lumbar disc herniations and to enable a clear distinction of surgical wound contamination arising from the normal skin microbiota, along with more detailed MLST analysis of isolates for the identification of particular STs that might be associated with this condition. It will also be valuable to determine the distribution of different phylogroups in lumbar skin preoperatively, and how these relate to the results from excised material, and further assess the effect of antimicrobial treatment upon pathological symptoms associated with the condition. Investigations into *P. acnes* pathogenesis and virulence mechanisms in associated lumbar disc infections are also warranted. 

## Figures and Tables

**Table 1:** MIC values (mg/L) for all *P. acnes* isolates recovered from discectomy tissue (*n* = 67). Values correspond to the total number of isolates with that specific MIC.

Antibiotic	MIC (mg/L)							
	≤0.03	0.06	0.12	0.25	0.5	1	2	≥4
Amoxicillin		31	36, 1*					
Ciprofloxacin						67, 1*		
Erythromycin	10	57, 1*						
Fusidic acid						11	56, 1*	
Rifampicin	67, 1*							
Tetracycline					67, 1*			
Vancomycin		1			23	43, 1*		
Trimethoprim							14	53, 1*
Gentamicin								67, 1*

*P. acnes NCTC 737.
